# Extra-embryonic endoderm cells derived from ES cells induced by GATA Factors acquire the character of XEN cells

**DOI:** 10.1186/1471-213X-7-80

**Published:** 2007-07-03

**Authors:** Daisuke Shimosato, Makoto Shiki, Hitoshi Niwa

**Affiliations:** 1 Laboratory for Pluripotent Cell Studies, RIKEN Center for Developmental Biology (CDB), 2-2-3 Minatojima-minamimachi, Chuo-ku, Kobe, Hyogo 650-0047, Japan; 2 Laboratory for Development and Regenerative Medicine, Kobe University Graduate School of Medicine, 7-5-1 Kusunokicho, Chuo-ku, Kobe, Hyogo 650-0017, Japan; 3 CREST (Core Research for Evolutional Science and Technology), Japan Science and Technology Agency, Honcho 4-1-8, Kawaguchi, Saitama 332-0012, Japan

## Abstract

**Background:**

Three types of cell lines have been established from mouse blastocysts: embryonic stem (ES) cells, trophoblast stem (TS) cells, and extra-embryonic endoderm (XEN) cells, which have the potential to differentiate into their respective cognate lineages. ES cells can differentiate *in vitro *not only into somatic cell lineages but into extra-embryonic lineages, including trophectoderm and extra-embryonic endoderm (ExEn) as well. TS cells can be established from ES cells by the artificial repression of *Oct3/4 *or the upregulation of *Cdx2 *in the presence of FGF4 on feeder cells. The relationship between these embryo-derived XEN cells and ES cell-derived ExEn cell lines remains unclear, although we have previously reported that overexpression of *Gata4 *or *Gata6 *induces differentiation of mouse ES cells into extra-embryonic endoderm in vitro.

**Results:**

A system in which GATA factors were conditionally activated revealed that the cells continue to proliferate while expressing a set of extra-embryonic endoderm markers, and, following injection into blastocysts, contribute only to the extra-embryonic endoderm lineage *in vivo*. Although the *in vivo *contribution is limited to cells of parietal endoderm lineage, Gata-induced extra-embryonic endoderm cells (gExEn) can be induced to differentiate into visceral endoderm-like cells *in vitro *by repression of *Gata6*. During early passage, the propagation of gExEn cells is dependent on the expression of the *Gata6 *transgene. These cells, however, lose this dependency following establishment of endogenous *Gata6 *expression.

**Conclusion:**

We show here that Gata-induced extra-embryonic endoderm cells derived from ES cells mimic the character of XEN cells. These findings indicate that Gata transcription factors are sufficient for the derivation and propagation of XEN-like extra-embryonic endoderm cells from ES cells.

## Background

During early mammalian development, the zygote cleaves several times and gives rise to embryonic and extra-embryonic lineages before implantation occurs. After compaction in the mouse embryo, the outer cells of the morula are epithelialized and become trophectoderm (TE), while the inner cells generate the pluripotent inner cell mass (ICM). The surface of the ICM adjacent to the blastocyst cavity differentiates into primitive endoderm (PrE), precursor cells of the extraembryonic endoderm (ExEn) lineage. PrE subsequently differentiates into visceral endoderm (VE) and parietal endoderm (PE) [1]. VE forms layers of columnar epithelial cells covering the epiblast and contributes to the visceral yolk sac, while PE migrates along the surface of the inner TE, secreting extracellular matrix to form the Reichert's membrane and contributes to parietal yolk sac as well [2]. These ExEn lineage cells are important in embryonic development, as nutritive supports and as a determinant of the anterior-posterior axis.

In early mouse development, the GATA family zinc-finger transcription factors *Gata6 *and *Gata4 *are specifically expressed in ExEn [3]. Expression of *Gata6 *starts at 3.5 *dpc *in ICM in a salt-and-pepper pattern, which is restricted to parietal endoderm at 7.0 *dpc *[4,5]. *Gata6 *knockout mice are embryonically lethal at 5.5 – 6.5 *dpc *due to defects in PrE formation and subsequent ExEn development [4,6]. *Gata6*-null embryonic stem (ES) cells fail to undergo VE differentiation *in vivo *and *in vitro *[6], and differentiation into ExEn does not occur, although *Gata4*-null ES cells can be induced to undergo epithelial differentiation by retinoic acid. [7]. This suggests that *Gata6 *function is required for early ExEn, including PrE, as well as for the development of both VE and PE.

Leukemia inhibitory factor (LIF) is required to maintain the pluripotency of mouse ES cells in conventional culture conditions; withdrawal of LIF causes ES cells to differentiate into PrE-like cells [8]. Overexpression of the POU family transcription factor *Oct3/4 *induced PrE-like differentiation with up-regulation of *Gata4 *[9], similar to the withdrawal of LIF, and overexpression of either *Gata4 *or *Gata6 *is sufficient to trigger the differentiation of ES cells into ExEn, which are similar to PE in morphology and gene expression pattern [10]. This indicates that ES cells possess the ability to differentiate into cells of the ExEn lineage, although they merely contribute to ExEn after injection into blastocysts[11].

Extra-embryonic endoderm (XEN) cells derived from blastocysts continuously propagate *in vitro*, while maintaining their ability to contribute to ExEn lineage cells after injection into blastocysts [12]. The morphology and expression of marker genes of XEN cells is similar to that of ES-derived PE cells induced by *Gata4 *or *Gata6*, suggesting that *Gata*-transfected ES cells may acquire XEN-like ExEn characteristics *in vitro*, although this has yet been confirmed.

Here, we report that an ExEn cell lines derived from mouse ES cells by the artificial activation of GATA factors acquire XEN-like properties. We characterized these cell lines, which we have designated gExEn cells, in comparison with embryo-derived XEN cells. gExEn cells express specific marker genes for ExEn and differentiate into both PE and VE *in vitro*. Moreover, their contribution *in vivo *is restricted to the ExEn lineage, as is that of XEN cells. Although GATA activation is continuously required for the propagation of gExEn cells during early passages, these cells can propagate without artificial activation of GATA in later passages, at which time endogenous GATA factors expression is induced and maintained. We show that GATA factors play a fundamental role in establishing and maintaining gExEn cells.

## Results

### Continuous propagation of ExEn cells induced from ES cells by ectopic expression of *Gata4 *or *Gata6*

By functional screening of transcription factors whose expression is upregulated after induction of differentiation in ES cells, we found that the GATA-family transcription factors *Gata4 *and *Gata6 *could induce differentiation toward the ExEn lineage [10]. Upon ectopic expression of *Gata4 *or *Gata6*, ES cells differentiated into dispersed refractive cells that resembled PE cells and expressed PE marker genes such as *Sparc *(*secreted acidic cysteine rich glycoprotein*) and *Plat *(*tPA; plasminogen activator, tissue*), indicating that activation of *Gata4 *and *Gata6 *is sufficient for inducing PE-like ExEn differentiation in ES cells.

**Figure 1:**
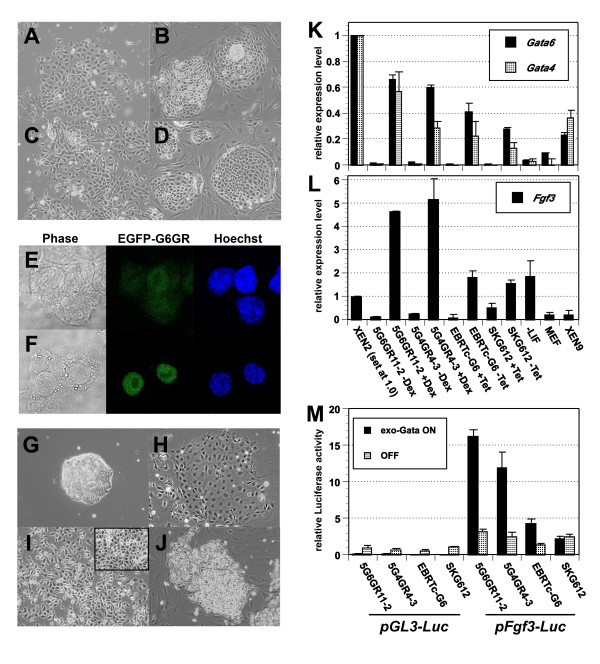
**Induction of gExEn cells from ES cells by inducible *Gata6 *or *Gata4 *expression systems**. (A-D) Morphology of differentiated SKG612 (A, B) and EBRTc-G6 (C, D) cells induced by withdrawal of Tc on gelatinized dishes (A, C) or under MEF culture conditions for 5 days (B, D). (E, F) Confocal microscopic image of ES cells with introduced *pCAG-EGFP-G6GR *2 hr after the addition of 70% EtOH, without (E) and with (F) Dex. The left panels show phase-contrast images, the center panels show localization of EGFP-G6GR monitored by EGFP fluorescence, and the right panels show nuclei stained with Hoechst33342. (G-J) Morphology of 5G6GR11-2 ES cells with or without Dex. 5G6GR11-2 ES cells were grown in the presence (G) or absence (H) of LIF for 5 days, or were treated with 100 nM Dex for 5 days on gelatinized dishes (I, high-density conditions in inset) or on MEF (J). (K) Expression levels of endogenous *Gata6 *and *Gata4 *in ES-derived ExEn cells 4 days after activation of exogenous *Gata *on gelatinized dish cultures or PrE induced by withdrawal of LIF for 5 days. XEN2 and XEN9 cells; derived from embryo and MEF-dependent propagation. MEF; MEF alone. All values were normalized relative to the level of *Gapdh *and plotted relative to levels of expression in XEN2 cells. (L) Expression levels of endogenous *Fgf3 *in ES-derived ExEn cells 4 days after activation of exogenous GATA on gelatinized dish cultures, or PrE induced by withdrawal of LIF for 5 days. XEN2 and XEN9 cells; derived form embryo and MEF-dependent propagation. MEF; MEF alone. All values were normalized relative to the level of *Gapdh *and plotted relative to levels of expression in XEN2 cells. (M) GATA-dependent enhancer activity of the element containing GATA-binding site of *pFgf3-luc *in ES-derived ExEn cells. Reporter plasmids were transfected into 5G6GR11-2 and 5G4GR4-3 ES cells followed by culture with (exo-GATA ON) or without Dex (OFF), or EBRTc-G6 and SKG612 ES cells followed by culture with (OFF), or without Tc (exo-GATA ON). Relative expression levels of *pFgf3-luc *(filled; exo-GATA ON, hatched; OFF) are shown. All results were normalized relative to the luciferase activities of *pCMV-RL *and plotted relative to the luciferase activities of *pGL3-luc *in Dex-non-treated 5G6GR11-2, set at 1.0.

XEN cells derived from blastocysts were recently reported to show very similar morphology to *Gata4 *or *Gata6 *induced PE cells derived from ES cells [12]. XEN cells were robust on mouse embryonic fibroblast (MEF) feeder layer or 70% conditioned medium (CM) from MEF for several passages. However, the ability of ExEn cells derived from ES cells to propagate following ectopic expression of *Gata4 *or *Gata6 *had not been determined. We therefore assessed the ability of *Gata6 *and *Gata4 *episomal transfectants with PE-like morphology to propagate in prolonged culture. We found, however, that these cells could be passaged fewer than three times in the culture conditions used for XEN cells (Table 1). Since the episomal expression system tends to become destabilized after induction of differentiation (Niwa, H., unpublished), we tested SKG612 [10] and EBRTc-G6 [13] ES cells, both of which carry integrated copies of tetracycline (Tc)-inducible *Gata6 *transgenes and differentiate into PE-like cells after induction of ectopic *Gata6 *expression following withdrawal of Tc (Fig. 1A or 1C). We found that, although SKG612-derived ExEn cells (Fig. 1B) could be passaged fewer than three times (Table 1), EBRTc-G6-derived ExEn cells (Fig. 1C) propagated continuously for more than 10 passages on MEF (Table 1), suggesting that these PE-like cells acquire XEN cell-like ability of proliferation.

**Table 1:** Culture conditions of three PE-like cells

**coating**	**MEF**		**Gelatin**	
**exo.Gata6**	**ON**	**OFF**	**ON**	**OFF**

MGZ5	±	-	-	-
SKG612	+	-	±	-
EBRTc-G6	+++	±	±	±
5G6GR	+++	+	+++	±

PE-like cells were derived from 5G6GR, EBRTc-G6 and SKG612 cells and grown with or without MEF, and in the presence or absence of Dex (5G6GR) or Tc (EBRTc-G6 and SKG612). Passage numbers: +++, more than 10; +, 3-10; ±, less than 3; - impossible to grow.

To further investigate the role of the GATA factors on ExEn differentiation and their XEN cell-like characteristics, we established another inducible activation system for the GATA factors in ES cells by introducing a chimeric transgene composed of full-length *Gata4 *or *Gata6 *and the human *glucocorticoid receptor *ligand-binding domain (*G4GR *and *G6GR*, respectively). Introduction of *pCAG-G4GR-IP *or -*G6GR-IP *into EB5 ES cells resulted in the establishment of the ES cell lines, 5G6GR (Fig. 1G) and 5G4GR (data not shown), respectively. GFP-tagged G6GR showed that, in the absence of dexamethasone (Dex), the chimeric transgene products were kept inactive in the cytoplasm (Fig. 1E), whereas, in the presence of Dex, they translocated into the nucleus (Fig. 1F), indicating that these chimeric molecules were properly regulated. Although parental EB5 ES cells had no morphological changes by the administration of Dex (data not shown), treatment with Dex altered the morphology of these 5G6GR or 5G4GR ES cells into dispersed, refractive and satellite type, reminiscent of PE cells. These cells, designated g6ExEn (Fig. 1I) and g4ExEn (data not shown) as found in episomal transfectants of *Gata4 *and *Gata6*, respectively, and were not similar to PrE induced by the withdrawal of LIF (Fig. 1H). Thus, these results indicated that the hormone-inducible GATA factors mimic the function of native GATA factors in ES cells.

g4ExEn and g6ExEn cells each had two distinct morphologies, depending on the culture conditions on gelatinized dishes. Dispersed refractive cells were observed in *Gata6 *or *Gata4 *transfectants under low-density culture conditions (Fig. 1I), whereas an epithelial sheet-type morphology was observed under high-density conditions (Fig. 1I, squares). In the presence of Dex, these cells could be expanded on MEF for more than 10 passages (Fig. 1J), similar to results for EBRTc-G6-derived ExEn cells (Table 1). These data suggested that gExEn cells acquire an ability to proliferate similar to that of XEN cells.

### High level of constitutive activation of GATA factors can substitute for the MEF requirement

Withdrawal of MEF was found to induce differentiation of XEN cells by reduction of *Gata4 *or induction of *Afp *(*alpha-fetoprotein*), a marker for VE in early embryos [12]. Both SKG612 and EBRTc-G6-derived ExEn cells showed limited capacity to propagate on gelatin-coated dishes in the absence of Tc, indicating an absolute requirement for MEF, as observed in XEN cells (Table 1 and Fig. 1A and 1C). In contrast, gExEn cells, derived from G4GR or G6GR ES cells induced by treatment with Dex, could be serially passaged on gelatinized dishes at about 1:40 dilution every 3 or 4 days (Fig. 1I). The culture period reached at least 40 passages for 4 g6ExEn lines (2-2, 2-3, 11-2, 11-3), and at least 10 passages for 2 g4ExEn lines (1-1, 4-3) on gelatinized dishes with no apparent senescence or reduction in viability (data not shown), indicating that they had lost their requirement for MEF for continuous propagation.

What is the molecular basis of the requirement of MEF for XEN cells? According to the original report by Kunath et al., MEF feeder layers can be substituted by the MEF-CM, indicating that one or more soluble factors secreted by MEF is required for XEN cells. If this signal is required for the transcriptional activation of *Gata6 *and/or *Gata4*, a constitutive supply of GATA factors from the transgenes beyond the threshold level may override the MEF dependency. Since we applied different chimeric transgenes in different inducible systems for activation of GATA factors and it was suggested that *Gata6 *and *Gata4 *possess cross- and auto-activation systems, simple measurement of the amount of transcripts for these transgenes, as well as the endogenous gene, was not a suitable indicator of the net GATA activity achieved in these transgenic ES cells. To achieve this, we used two different approaches; (1) transcriptional quantification of the endogenous GATA target genes, and (2) measurement of the activity of GATA-dependent reporters. When the *luciferase *(*luc*) reporter carrying the *Gata6 *promoter and the first intron (*pGata6-luc*), where the auto-regulatory elements are found in other *Gata family genes *[14], was introduced in 5G6GR and 5G4GR ES cells, it was significantly activated within 24 hours in the presence of Dex in both cell lines, strongly suggesting the direct activation of this reporter by GATA6 and GATA4 ' [see additional file 1]'. We quantified endogenous *Gata6 *in ES cells carrying various types of inducible *Gata6 *transgenes and found that the expression levels of *Gata6 *in G6GR and G4GR cells with Dex were high, that of EBRTc-G6 with Tc was moderate, and that of SKG6 with Tc was low, but still significantly higher than that of PrE cells induced by withdrawal of LIF (Fig. 1K). The expression levels of *Gata6 *were varied in two XEN cell lines established from blastocysts (XEN2 and XEN9), but the ranges of the expression levels were comparable to those of ES-derived ExEn cells (Fig. 1K). Similar results were obtained for *Gata4 *expression, suggesting the presence of its auto-and cross-regulation by GATA factors. As the additional indicator, we chose *Fibroblast growth factor (Fgf)-3 *because the direct regulation of its promoter by GATA4 has been reported [15]. We confirmed that the activation of the *pFgf3-luc *and *pFgf3-tk-luc *reporter was comparable to that of *pGata6-luc *at 24 hours after induction of exogenous GATA activities (Fig. 1M and ' [see additional file 1]') and found that either the activities of *pFgf3-luc*/*pFgf3-tk-luc *or the transcription levels of endogenous *Fgf3 *at 4 days after induction of exogenous GATA activities were consistent with the hierarchy of the *Gata6 *expression levels in these ExEn cells (Fig. 1L and 1M), indicating that the induced GATA activity is highest in 5G6GR and 5G4GR, moderate in EBRTc-G6 and lowest in SKG612. This indicates a tight relationship between the ability to self-renew in the absence of MEF and the induced activity of constitutive GATA factors independent to exogenous signal, suggesting a role for the maintenance of GATA activity at a high level in the propagation of XEN cells under feeder-free conditions.

### Expression of ExEn marker genes in gExEn cells

**Figure 2:**
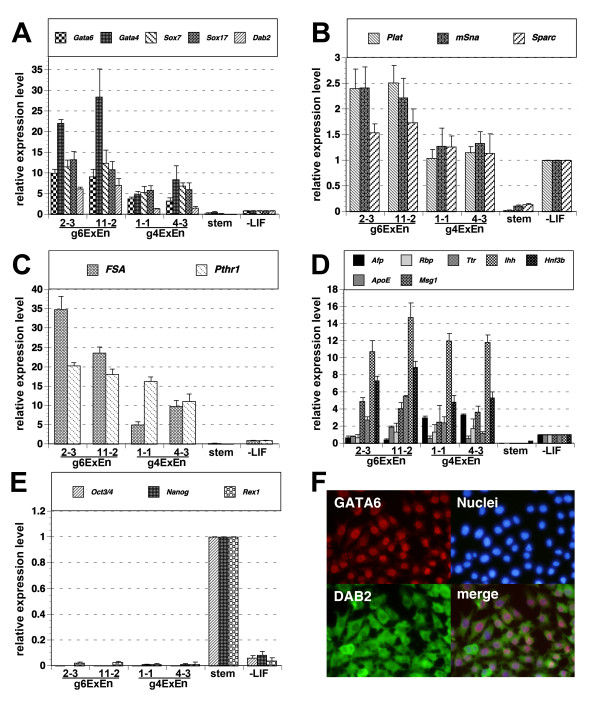
**Marker gene expression of gExEn cells**. (A-E) Q-PCR analysis of gene expression in Dex-treated gExEn cells after 10 passages. ExEn markers (A), PE markers (B and C), VE markers (D) and stem cell markers (E). Relative expression levels of the indicated marker genes in two independent clones of g6ExEn (2-3 and 11-2) and g4ExEn (1-1 and 4-3) and in 5G6GR ES cells, in the presence and absence of LIF for 5 days, are shown. All results were normalized relative to the level of expression *Gapdh *and plotted relative to expression levels in 5G6GR-derived PrE without LIF (A-D) or that in 5G6GR ES cells (E). (F) g4ExEn4-3 were stained with anti-GATA6 (red), anti-DAB2 (green), and nuclear staining by Hoechst33342 (blue). The lower right panel shows marginal images.

To investigate the detailed characteristics of g4ExEn and g6ExEn cells, we analyzed marker gene expression in passage 10 cells by quantitative PCR (Q-PCR). We chose *Gata4 *[16], *Gata6 *[4], *Sox7 *and *Sox17 *[17], and *Disabled homolog 2 *(*Dab2*; [18]) as ExEn markers, which are markers for ExEn, and found that all were induced in gExEn cells at much higher levels than in PrE from ES cells induced by the withdrawal of LIF (Fig. 2A). The expression levels of these markers were about 2-fold higher in g6ExEn than in g4ExEn cells, which might reflect functional differences between *Gata6 *and *Gata4 *as previously reported, in which GATA6 is an upstream regulator of *Gata4 *while GATA4 is a negative regulator of *Gata6 *[6,7].

The level of expression of the PE markers *Plat *[19], *Snail *[20], and *Spar*c [21] in g6ExEn and g4ExEn cells was similar to that in PrE cells (Fig. 2B), whereas expression of *Follistatin *(*Fst*; [22]) and *Parathyroid hormone receptor 1 *(*Pthr1*; [23]) was much higher in g6ExEn and g4ExEn cells than in PrE cells (Fig. 2C).

The VE markers *Afp *[24], *Foxa2 *(*Hnf3b*; [25]), *Indian hedgehog *(*Ihh*; [26]), *Transthyretin *(*Ttr*; [27]), *Retinol-binding protein *(*Rbp*; [28]), *Apolipoprotein E *(*ApoE*; [29]), and *Cbp/p300-interacting transactivator with Glu/Asp-rich carboxy-terminal domain 1 *(*Cited1/Msg1*; [30]) were also induced to an equivalent or higher extent in g6ExEn and g4ExEn cells than in PrE cells (Fig. 2D). In contrast, the pluripotent cell markers *Oct3/4 *(*Pou5f1*), *Nanog *and *Zfp42/Rex1*, as well as the trophectoderm markers *Cdx2*, *Hand1 *and *Psx1 *were hardly detected in gExEn cells (Fig. 2E and data not shown). These data fit the marker gene expression profile of XEN cells reported previously.

Immunohistochemical analysis showed that virtually all g4ExEn4-3 express GATA6 in the nucleus (Fig. 2F, red) and DAB2 in the cytoplasm (Fig. 2F; green), similar to embryo-derived XEN cells (data not shown), indicating that gene expression profile of gExEn cells is homogeneous, as judged by their morphology.

### g4ExEn and g6ExEn cells contribute to the parietal endoderm lineage *in vivo*

To determine the *in vivo *differentiation potential of gExEn cells, we performed chimera analysis with cell lines carrying *pCAG-EGFP-IZ*, 5G4GR-GFP and 5G6GR-GFP. These cell lines differentiated into gExEn and showed strong EGFP expression, with or without Dex treatment, which was sufficient for the detection of their progeny cells at the single-cell level in chimeric embryos.

**Figure 3:**
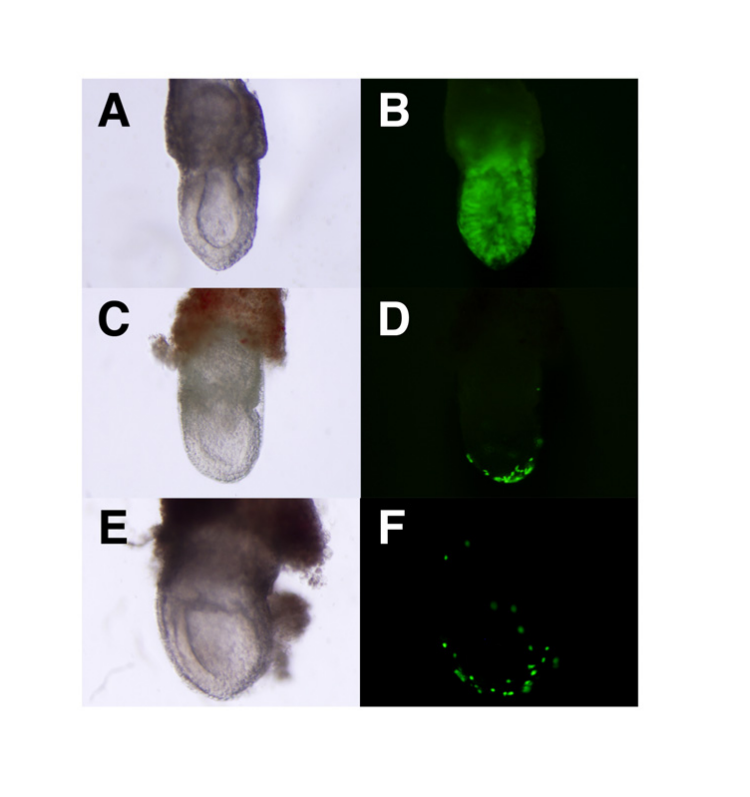
**Contribution of gExEn cells to ExEn lineage *in vivo***. (A, B) 8.5 dpc chimeric embryos derived from 5G6GR-GFP ES cells. 5G6GR-GFP ES cells, kept in undifferentiated state without Dex, give rise to embryonic chimeras. The 8.5 dpc chimeric embryos with g6ExEn-GFP (C, D) derived from 5G6GR-GFP ES cells or g4ExEn-GFP cells (E, F) derived from 5G4GR-GFP ES cells, cultured in the presence of Dex for the activation of GATA-GR after several passages, contributed only to the distal parietal yolk sac.

When 5G6GR-GFP ES cells, without Dex treatment, were injected into wild-type C57Bl6/6J blastocysts and transferred into the uteri of pseudopregnant mice, they generated 16 chimeric embryos, in which the EGFP-positive cells contributed only to the embryonic portion and were never found in the extra-embryonic lineage (Fig. 3A, B and Table 2). In contrast, when g4ExEn-GFP and g6ExEn-GFP cells derived from the Dex-treated ES cell lines 5G6GFP-GR and 5G4GFP-GR, respectively, were injected after several passages, they contributed exclusively to the extra-embryonic yolk sac, with a scattered pattern, a feature of PE *in vivo*, in the chimeric embryos (Fig. 3C–F and Table 2): This result was previously observed for embryo-derived XEN cells [12]. However, in all chimeras, the contribution of the gExEn cells was restricted to the parietal yolk sac. A similar tendency was found for XEN cell chimeras, in which XEN cells in VE were observed in only 1 in 50 chimeras [12]. These findings indicate that g4ExEn and g6ExEn cells derived from ES cells by artificial activation of *Gata4 *or *Gata6 *have the potential to contribute to only PE *in vivo*.

**Table 2:** Blastocyst injection data

**line name**		**Number of deciduas**	**Number of embryos**	**Number of embryos**	
				**embryonic**	**ExEn**
ES	(G6GR-Dex)	36	25(70%)	16(44%)	0
					
g6ExEn	2-2	6	6	0	2
	2-3	25	10	0	8
	11-2	79	35	0	14
	11-4	15	4	0	1

	total	125	54(43%)	0	25(20%)
					
g4ExEn	1-1	13	8	0	4
	4-3	18	9	0	6
	4-5	13	5	0	2

	total	54	22(41%)	0	12(22%)

4 g6ExEn cell clones (2-2, 2-3, 11-2, 11-4) and 3 g4ExEn cell clones (1-1, 4-3, 4-5) were used to generate chimeras by blastocyst injection.

### Activation of exogenous Gata factors is required for propagation of gExEn cells in the early passage period

**Figure 4:**
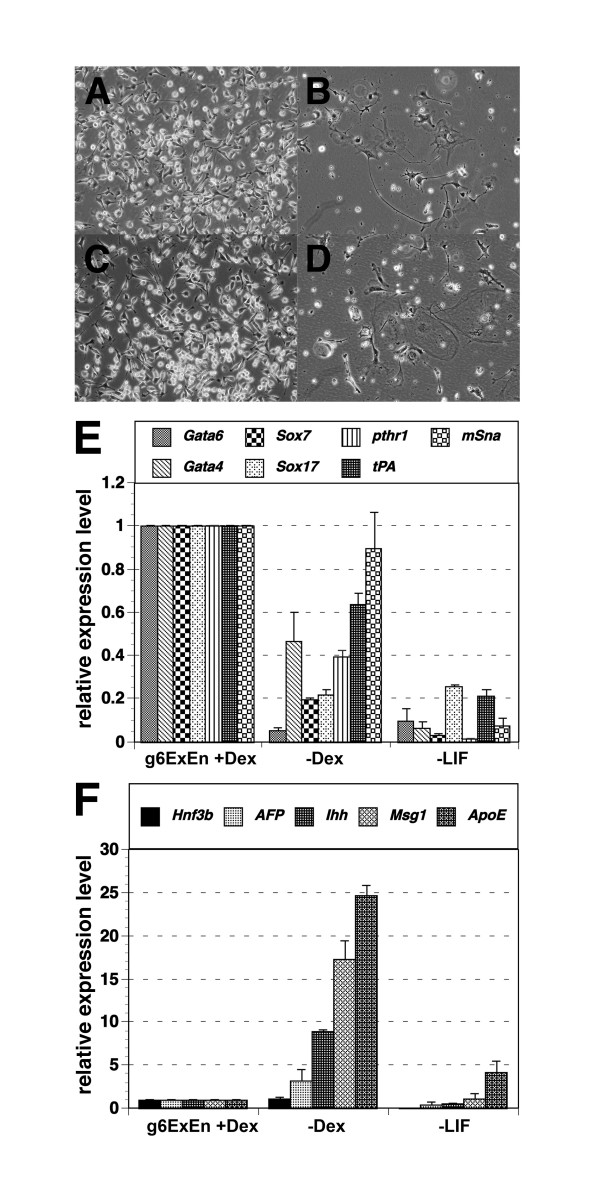
**Effect of the extinction of exogenous GATA activity in gExEn cells**. (A-D) Photomicrographs of g6ExEn11-2 (A, B) and g4ExEn4-3 (C, D) cells cultured with (A or C) or without (B or D) Dex for 4 days after 2 passages in the presence of Dex. (E, F) Q-PCR analysis of PE or VE marker gene expression in g6ExEn11-2 cells, with or without Dex. Withdrawal of Dex after 2 passages in the presence of Dex decreased expression of a set of PE marker genes (E), while expression of VE marker genes increased in parallel (F). All results were normalized relative to expression of *Gapdh *and plotted relative to the expression level in Dex-treated g6ExEn11-2 cells.

Although in the presence of Dex g4ExEn and g6ExEn cells are robust, even in the absence of MEF (Fig. 4A, C), these cells gradually ceased to propagate and their morphology became flatter after withdrawal of Dex (Fig. 4B, D). Similar changes were observed in gExEn cells cultured on MEF, suggesting that continuous activation of Gata-GR is absolutely required for the propagation of gExEn cells.

To confirm the status of gExEn cells with or without exogenous GATA activity, we assayed expression of several VE and PE marker genes by Q-PCR. Expression of endogenous *Gata4 *and *Gata6 *was reduced by inactivation of GATA6-GR in g6ExEn cells following withdrawal of Dex (Fig. 4E). In addition, expression of the ExEn marker genes *Sox7 *and *Sox17*, and the PE marker genes *Plat*, *Snail *and *Pthr1 *were decreased after withdrawal of Dex, whereas the VE marker genes *Afp Hnf3b*, *Ihh*, *Ttr*, *Rbp*, *ApoE *and *Cited1 *were increased in parallel (Fig. 4E, F). These findings are similar to the gene expression profile in differentiated XEN cells induced by the withdrawal of MEF, in which decreasing expression of *Gata4*, and *Gata6 *and several PE marker genes, and increasing expression of VE markers including *Afp*, was observed [12]. Therefore, withdrawal of exogenous GATA6 activity induces differentiation of gExEn cells, indicating that maintenance of GATA6 activity is required for propagation of gExEn cells.

### Establishment of endogenous Gata expression restores dependency on exogenous Gata activity

**Figure 5:**
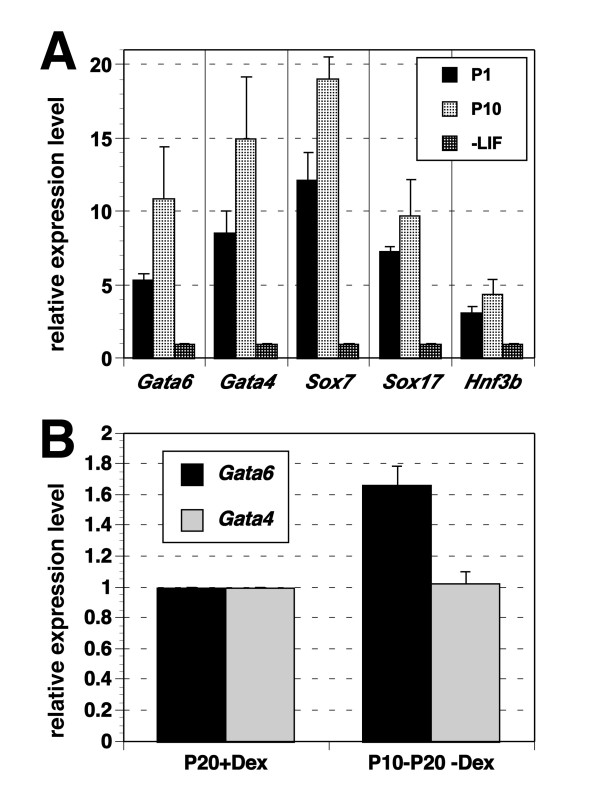
**Expression of endogenous *Gata4 *and *Gata6 *in late-passage gExEn cells**. (A) Marker gene expression in late passage: g6ExEn cells expressed endogenous *Gata4 *and *Gata6 *slightly higher than in early passage g6ExEn cells. All results were normalized relative to expression of *Gapdh *and plotted relative to the expression level in 5G6GR-derived PrE without LIF for 5 days. (B) After removal of Gata6-GR activity by withdrawal of Dex from passage10 to passage20 (P10-P20), the expression levels of endogenous *Gata4 *and *Gata6 *were maintained as same amount level in the presence of Dex culture condition (P20). All results were normalized relative to expression of *Gapdh *and plotted relative to the expression level in P20 g6ExEn cells.

Interestingly, over about 5 passages, the dependency of gExEn cells on exogenous Gata activity was gradually lost and they became able to propagate without Dex. Analyses of marker gene expression in these late passage gExEn cells revealed that the expression levels of endogenous *Gata4 *and *Gata6 *were slightly higher than in early passage gExEn cells (Fig. 5A). In contrast to early passage gExEn cells (Fig. 4E), the expression levels of endogenous *Gata4 *and *Gata6 *were maintained after removal of GATAF6-GR activity by withdrawal of Dex (Fig. 5B). These data indicated that the positive auto-regulatory loop that maintains expression of endogenous Gata factors was gradually established during cell culture.

**Figure 6:**
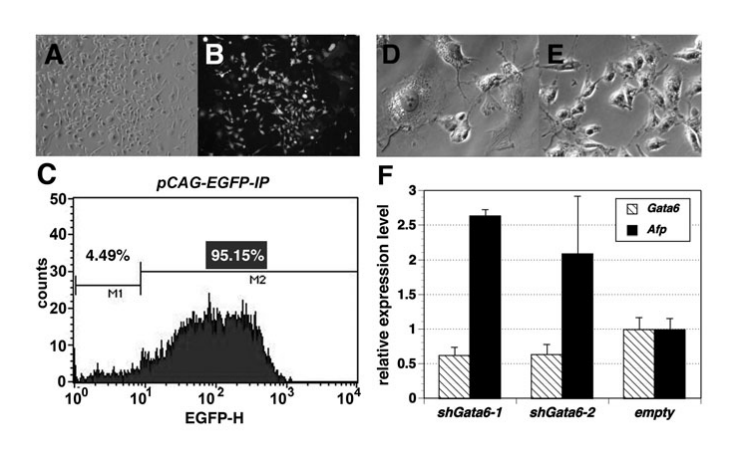
**Knock-down of *Gata6 *in late-passage gExEn cells**. (A-C) Efficient transfection of g6ExEn cells by EGFP expression vector. After drug selection, almost all transfectants showed GFP expression microscopically (A: phase-contrast image, B: fluorescent image for EGFP), which was confirmed by FACS analysis for EGFP fluorescence (C). (D, E) Morphology of g6ExEn cell line, 1D3, transfected with the *Gata6 *silencing vector *pSil-G6 *(D) or empty vector (D) after 5 days transfection. (F) Expression level of *Gata6 *and *Afp *in *Gata6*-silenced 1D3 cells by Q-PCR at day 5. Results were normalized relative to expression of *Gapdh *and plotted relative to expression level in 1D3 cells transfected with *pSil-H1puro*.

To further determine whether *Gata6 *activity is required to maintain propagation of late passage gExEn cells, we performed a loss-of-function assay by silencing *Gata6 *expression using a short-hairpin RNA-mediated knockdown strategy. *pSil-H1puro *expresses short hairpin RNA under the control of the mouse *H1-RNA *gene promoter. A vector targeting *Gata6*, *pSil-shG6*, was transfected into Dex-independent puromycin sensitive g6ExEn cell line, 1D3, and the transfectants were cultured for 48 hr under puromycin selection and then analyzed for ExEn marker expression by quantitative PCR. Transfection efficiency in 1D3 cells was monitored by transient expression of EGFP using *pCAG-EGFP-IP *vector transfected by the same protocol (Fig. 6A, B). FACS analysis showed that about 95% of the cells were EGFP-positive (Fig. 6C).

The level of expression of endogenous *Gata6 *in the 1D3 cells transfected with *pSil-shG6 *was about 50% of that in control cells transfected with *pSil-H1puro *(Fig. 6F). After puromycin selection, the control cells exhibited no morphological changes (Fig 6E), whereas the 1D3 cells stopped propagating and showed altered morphology, similar to that observed during VE-like differentiation induced by withdrawal of Dex during the early passage period (Fig. 6D), including a 3-fold upregulation of *Afp *relative to control cells (Fig. 6F). These data suggested that *Gata6 *is absolutely essential for the propagation of gExEn cells.

## Discussion

The systematic *in vitro *differentiation of ES cells represents a powerful tool for analyzing the molecular mechanisms controlling pre-implantation development [31]. However, careful comparison of events observed *in vitro *and *in vivo *is required to use this model system properly. We have characterized gExEn cells generated *in vitro *from ES cells by the artificial activation of GATA factors and confirmed that they mimic the characteristics of XEN cells. gExEn cells can be propagated continuously on gelatinized dishes by constitutive activation of exogenous GATA activity, independent of the MEF-derived signal, and contribute to ExEn in chimeric embryos, as do XEN cells. Although the *in vivo *contribution of gExEn cells is limited to PE, these cells differentiate *in vitro *into cells morphologically and genetically similar to VE cells. In addition, we confirmed that *Gata6 *is important for the propagation of gExEn cells. These data clearly indicate that ectopic and continuous activation of GATA4 or GATA6 is sufficient to trigger proper differentiation of ES cells into the ExEn lineage.

Lineage specification by tissue-specific transcription factors is a key step in development. In mouse blastocysts, there are three cell lineages, ICM, TE and ExEn, with various cell lines derived from each. To date, several ExEn cell lines have been described, including the rat yolk sac carcinoma line L2 [32], the RE1 line from a rat blastocyst [33], parietal endoderm cells (PEC; [34]) and XEN cells [12] from mouse blastocysts. Of these, XEN cells are regarded as the best model of ExEn development *in vitro *because of their origin and characteristics *in vivo *after injection into blastocysts. However, the molecular mechanisms of derivation and propagation of XEN cells have not yet been analyzed, although the functions of GATA factors in XEN cells were suggested by both gain- and loss-of-function studies *in vitro *and *in vivo*, showing that *Gata4 *and *Gata6 *were necessary and sufficient to commit cells to the ExEn lineage [4,6,10].

We have clearly shown here that GATA factors play a central role in the induction and maintenance of gExEn cells. Although transient induction of ectopic GATA4 or GATA6 activity is sufficient to induce differentiation of ES into gExEn cells (data not shown), inactivation of the exogenous GATA activity in the early passage period prevented their propagation and the induction of terminal differentiation. In the late passage period, gExEn cells were freed from their dependency on exogenous GATA activity, but still had a tight requirement for endogenous GATA expression. These findings indicate that gExEn cell propagation is dependent on GATA factors, and that this may also be applicable to embryo-derived XEN cells.

Although XEN cells derived from blastocysts grow robustly on MEF or in medium supplemented with 70% MEF-CM, which contains many unknown factors, we found that gExEn cell propagation is dependent only on the high level of the induced activation of GATA4 or GATA6, without any exogenous factors. In contrast, EBRTc-G6-derived ExEn cells, which showed weaker expression of endogenous *Gata6 *following induction of exogenous *Gata6 *than gExEn cells, mimic the MEF-dependency of XEN cells. Since the requirement for MEF can be satisfied by high-levels of GATA factors, the soluble factors contained in MEF-CM may activate the expression of endogenous GATA factors, as we hypothesized. To date, we have tested the activity of several candidate soluble factors to substitute the role of MEF feeders, but neither FGF3 [15], a soluble factor abundantly expressed in PE, nor parathyroid hormone-like peptide (Pthih/PTHrP; [23]) secreted from TE, the ligand of the PTHrP receptor expressed in PE, can substitute for activation of GATA-GR fusion protein by Dex to maintain the propagation of gExEn cells under feeder-free conditions (data not shown). In contrast, as suggested for the possible involvement of the LIF signal for XEN cell maintenance [12], the addition of LIF in the culture of gExEn cells enhanced their propagation (data not shown). The relationship between the activities of GATA factors and the soluble factor(s) in MEF-CM will be tested using the *in vitro *model system with gExEn cells and XEN cells.

The role of MEF-derived soluble factor(s) might not be restricted in the transcriptional activation of endogenous *Gata6 *and *Gata4*. It has been reported the post-translational modification of GATA4 is important to acquire full transcriptional activity [35]. According to this report, acetylation of GATA4, which might be mediated by p300, increases its DNA-binding, resulting enhancement of its transcriptional activity. It is also possible that this pathway is regulated by MEF-derived factor(s). Efficient maintenance of ExEn cells without MEF by activation of GATA4GR or GATA6GR might reflect their ability to compensate for both signal dependencies on transcription and post-translational modification by an unexpected effect of the fusion to the GR ligand binding domain.

gExEn cells express many ExEn marker genes, including those specific for VE and PE. gExEn cells have the potential to contribute to PE *in vivo *in chimeras, as do XEN cells, indicating that activation of *Gata4 *or *Gata6 *is sufficient to induce proper differentiation of XEN-like cells from ES cells. However, as is the case for XEN cells, gExEn cells exhibit a strong bias to contribute to PE in chimeric embryos. Indeed, *Gata6 *is required for VE formation; *Gata6*-null ES cells fail to differentiate into VE on the surface of embryoid body [4,6,7]. During Dex withdrawal-induced differentiation of early passage gExEn cells *in vitro*, their morphology became flattened with ruffled membranes, reminiscent of VE following upregulation of VE markers, as found in XEN cells. Blastocyst injection showed, however, that PrE and nascent VE cells directly isolated from embryos contributed mostly to PE, indicating that PrE or VE dissected from ICM or epiblast tends to become PE [23]. Interestingly, Casanova and Grabel [36] reported that VE-like cells derived from the embryoid bodies of F9 embryonal carcinoma cells maintain the VE phenotype on the surface of EB or gelatin-coated dextran beads but lose it rapidly under monolayer conditions, with repression of the VE marker *Afp *and activation of the PE marker *Plat*. Therefore, the bias of gExEn and XEN cells to the PE phenotype may be due to 2-dimensional culture conditions, which are not permissive for maintenance of the VE phenotype.

As previously shown, we found that artificially-expressed *Gata4 *or *Gata6 *activated both endogenous *Gata4 *and *Gata6 *[10] to maintain the propagation of gExEn cells. After 5 passages, however, gExEn cells gradually acquire the ability to propagate without activation of exogenous Gata factors. Such weaning from exogenous GATA activity may be achieved by locking the auto-regulatory positive feedback loop between endogenous *Gata4 *and *Gata6*. This may mimic the situation *in vivo*, where transient exogenous signals activating the expression of Gata factors are required to generate the mature ExEn cell population that propagates continuously as it expands along the yolk sac. It is also possible to regard this phenomenon as an artificial condition generated by continuous activation of GATA factors at high levels. In any case, since the embryo-derived XEN cells never proliferate without MEF, the balance of the transcription factors in these cell lines should be different, and a global comparison of their transcriptomes will provide a cue to solve the structure of the transcription factor network including *Gata4 *and *Gata6 *in ExEn cells.

## Conclusion

We have succeeded in the establishment of ExEn cell lines, that have the same character of XEN cells derived from embryos, from ES cells by the constitutive activation of ExEn specific transcription factor, GATA4 and GATA6.

Establishment of gExEn cells, as with TS cells [37], from ES cells confirmed that the two differentiation events in mouse pre-implantation development could be mimicked by the *in vitro *activation of lineage-specific transcription factors. This model can be regarded as a powerful tool for investigating the transcriptional network transition from pluripotent stem cells to lineage-restricted cells.

## Methods

### Plasmid construction

DNA manipulations were performed by standard procedures [38]. Full details of plasmid constructions are available on request.

To generate *Gata6-GR *or *Gata4-GR *chimeric genes (designated *G6GR *and *G4GR*, respectively), the cDNA fragment encoding the ligand-binding domain (LBD) of the human *glucocorticoid receptor *(*GR*) was amplified by PCR, using the oligonucleotide primers, 5'-ACCATGGAAAATCCTGGTAACAAAACA-3' and 5'-ATGCGGCCGCTCACTTTTGATGAAACAGAAG-3', which contained NcoI and NotI restriction sites (underlined), respectively. The fragment was ligated into the NcoI and NotI sites of *pCAG-cHA-IP *(a derivative of *pCAG-IP*: [39]), resulting in the generation of *pCAG-chGR-IP*. Full-length mouse *Gata4 *and *Gata6 *cDNAs were PCR amplified from *pCAG-Gata4-IP *and *pCAG-Gata6-IP*, respectively, using the oligonucleotide primers, 5'-CCTCGAGCTTGGGGCGATGTACCAA-3' and 5'-AATCATGACCGCGGTGATTATCTCCCCATG-3' for Gata4, and 5'-TTCTCGAGCAGCCGGAGGAAATGTACC-3' and 5'-AATCATGAGGGCCAGAGCACACCAAGAATC-3' for Gata6, each set of which contained XhoI and BspHI restriction sites (underlined) [10], and inserted into the XhoI and NcoI sites of *pCAG-chGR-IP*, generating *pCAG-G6GR-IP *and *pCAG-G4GR-IP*, respectively.

To visualize nuclear translocation of the chimeric GR protein, *pCAG-EGFP-G6GR *was constructed by in-frame insertion of *EGFP *upstream of *G6GR*.

### Gene silencing

We used pSilencer 3.1 H1 puro vector (*pSil-H1puro*; Ambion) for gene silencing. Specific hairpin-forming inserts containing the 19-mer siRNA target sequence of *Gata6*, 5'-TGCGTTGCAGCAATCAGTG-3' (N19) [40], a linker sequence (5'-TAGTGAAGCCACAGATGTA-3'), and six thymidines as a termination signal were generated using a pair of nucleotides, 5'-GGATCCTGAGCGA-(senseN19)-(linker)-(antisenseN19)-GTGCCTATTTTTTGGAAA-3', which included a BamHI site (underlined), and 5'-AAGCTTTTCCAAAAAATAGGCAC-(senseN19)-(TACATCTGTGGCTTCACTA-linker)-(antisenseN19)-TCGCTCAG-3', which included a HindIII site (underlined). After annealing these oligonucleotides, the resulting double-stranded fragments were ligated into the BamHI and HindIII sites of *pSil-H1puro*, resulting in the generation of *pSil-shG6 *and the hairpin-forming inserts were sequenced using an ABI 3130 xl genetic analyzer.

For the transfection of *pSil-shG6*, we established another 5G6GR cell lines carrying *pCAG-Gata6GR-IRES-HisD *expression vector, designated for 1D3. 1D3 cells can differentiate to ExEn and propagate in the presence of Dex condition as same as 5G6GR cells.

### Cell culture and transfection

All ES cells were cultured on gelatin-coated dishes in the absence of feeder cells in Glasgow minimal essential medium (GMEM; Sigma) supplemented with 10% fetal calf serum (FCS), 1 mM sodium pyruvate (Invitrogen), 10^-4 ^M 2-mercaptoethanol, 1× non-essential amino acids (Invitrogen) and 1000 U/ml of LIF.

Transfection of the expression vectors into ES cells was performed as described using Lipofectoamine 2000 (Invitrogen) [10]. 5G6GR and 5G4GR ES cells were generated by random integration of the linearized Gata6-GR and Gata4-GR expression vectors, respectively, into E14tg2a-derived EB5 ES cells, in which one endogenous *Oct3/4 *allele is disrupted by a blasticidin resistance gene [41].

For Gata-GR activation, 100 mM dexamethasone (Dex: Sigma) was added to the culture of 5G6GR or 5G4GR ES cells, with the resulting ExEn cells designated g6ExEn and g4ExEn cells, respectively. These gExEn cells were cultured using the same conditions as ES cells, except for withdrawal of LIF. The gExEn cells, which can propagate without Dex, were transiently transfected with gene silencing vectors using the same method as for ES cells.

### Luciferase reporter assay

For the construction of *pFgf3-luc *vector, fragment of DNA encompassing 1.7 kb of sequence immediately 5' of the *Fgf3 *coding region containing GATA binding site [42] was PCR amplified from BAC containing 5' sequence of *Fgf3 *genomic region using the oligonucleotide primers, 5'-AAAGGATTCAGATGCCCTCTGGAT-3', which included a BamHI site (underlined), and 5'-TTTGCCGGCTCGACTGTGGCTA-3', which included a NaeI site (underlined), and inserted into the BglII and HIndIII (Blunted) sites of *pGL3 *(Promega).

For transfection of reporter plasmids, 1 × 10^4 ^cells were seeded in each well of a 96-well plate and incubated with 0.33 μg reporter plasmid and 0.33 ng of the internal control plasmid pRL-CMV, together with Lipofectoamine 2000 (Invitrogen), following the manufacturer's protocol. Luciferase assays were performed 24 hours later using a Dual-luciferase assay kit (Promega).

### Derivation and culture of XEN cells

Following overnight culture of 3.5 dpc C57Bl/6J blastocysts in KSOM (Specialty Media), the blastocysts were incubated at 37°C for 5 min with 0.5% *S. griseus *protease (Pronase; Sigma) to remove the zona pellucida, plated on 48-well plates coated with mouse embryonic feeder cells, and cultured in RPMI1640 (Gibco) containing rhFGF4 (25 ng/ml, Wako), following the conditions as described by Kunath et al. [12]. After 7 days, XEN-like cells were passaged 1:1 onto new MEF in 4-well plates; after another 7 days, two lines of XEN cells (XEN2 and XEN9) were passaged in FGF4-free media.

### Production of chimeric embryos

To visualize the *in vivo *contribution of gExEn cells, 5G6GR-GFP and 5G4GR-GFP ES cells were established by introducing constitutive EGFP expression vector (*pCAG-EGFP-IZ*) into 5G6GR and 5G4GR ES cells, respectively. To obtain chimeric embryos, ES and g6ExEn-GFP or g4ExEn-GFP cells were injected into C57Bl/6J blastocysts (2–3 cells per blastocyst), followed by transfer into the uteri of pseudopregnant ICR mice.

Embryos were dissected at 8.5 *dpc *and fluorescent signals were detected using an Olympus SZX12 fluorescent dissecting microscope and captured with an Olympus DP70 cooled color digital (CCD) camera.

### RNA preparation and real-time PCR

Total RNA was prepared using TRIzol reagent (Invitrogen) according to the manufacturer's instructions. First strand cDNA was synthesized from 1 μg of total RNA in 40 μl containing oligo-dT primers using a ReverTra Ace first-strand synthesis kit (Toyobo). Real-time PCR was performed with the ExTaq cyber green supermix (Takara) using an iCycler System (Bio-Rad). The amount of target RNA was determined from the appropriate standard curve, and was normalized relative to the amount of *Gapdh *mRNA. Sequences of primers for QPCR are listed on Table 3. *Gata6 *or *Gata4 *primer pairs were designed to amplify 3' untranslated regions, and thus to detect only endogenous transcripts.

**Table 3:** Primer Sequences for Q-PCR

*Gene*	Primer	Sequence	*Gene*	Primer	Sequence
*Gata6*	F	GAGCTGGTGCTACCAAGAGG	*Plat*	F	GATGACAGGGAGATGCCAAC
	R	TGCAAAAGCCCATCTCTTCT		R	CTTGTCCCCAGTGCAAACTT
*Gata4*	F	CCCTTCCCTCTTCAAATTCC	*mSna*	F	GCTGTGTTGGAAACGGAGTT
	R	CTTTTCCAGAGCTCCACCTG		R	CATGTGGGTTCTGACTGGTG
*Sox7*	F	GCTCCTGCTTTTGGTGTAGC	*SPARC*	F	GTTCCTGCTTGGCTCTCTTG
	R	GTCCTTGGGCAGTCATTCAT		R	CCTTGAGGGAGGTAGGGAAG
*Sox17*	F	GAGGGCCAGAAGCAGTGTTA	*Follistatin*	F	ACCTGAGAAAGGCCACCTG
	R	AGTGATTGTGGGGAGCAAGT		R	AGCTTCCTTCATGGCACACT
*Dab2*	F	TCTCAGCCTGCATCTTCTGA	*Pthr1*	F	AGGACGACGGCTTCCTTAAT
	R	GAGCGAGGACAGAGGTCAAC		R	TTGTCTTCCTGGTCCAGTCC
*AFP*	F	TCCAGAAGGAAGAGTGGACAA	*Oct3/4*	F	CACGAGTGGAAAGCAACTCA
	R	GCAGACTAGGAGAAGAGAAATAGTTGA		R	AGATGGTGGTCTGGCTGAAC
*ApoE*	F	GGTTCGAGCCAATAGTGGAA	*Nanog*	F	CACCCACCCATGCTAGTCTT
	R	TATTAAGCAAGGGCCACCAG		R	ACCCTCAAACTCCTGGTCCT
*RBP*	F	GAACTTCGACAAGGCTCGTTTCTCGG	*Rex1*	F	GAGTTCGTCCATCTAAAAAGGGAGG
	R	ATCCAGTGGTCATCGTTTCCTCGCT		R	TCTTAGCTGCTTCCTTGAACAATGCC
*Ihh*	F	GGCCTGGGATTGTGACTTTA	*Cdx2*	F	AGGCTGAGCCATGAGGAGTA
	R	CTGCAGGGAAGGTCATGTTT		R	CGAGGTCCATAATTCCACTCA
*Hnf3b*	F	CCCTGCTAGCTCTGGTCACT	*Hand1*	F	CCCCTCTTCCGTCCTCTTAC
	R	ACAGATCACTGTGGCCCATC		R	CTGCGAGTGGTCACACTGAT
*Msg2*	F	ATGCCAACCAGGAGATGAAC	*Psx1*	F	GAATTGGTTTCGGATGAGGA
	R	AGGATGCAGGTTGAAGGATG		R	GTGGCTCAGAAGAAGCCATC
*Ttr*	F	CTCACCACAGATGAGAAG	*Gapdh*	F	ACCACAGTCCATGCCATCAC
	R	GGCTGAGTCTCTCAATTC		R	TCCACCACCCTGTTGCTGTA

F, forward; R, reverse

### Immunostaining

Cells were fixed with 10% formaldehyde for 5 min at room temperature, washed with PBS containing 0.5% Triton X-100 for 10 min at room temperature, and incubated with rabbit anti-Gata6#1 antibody that we raised by immunization with GST-GATA6 fusion protein followed by affinity purification and anti-Disabled-2 (p96) monoclonal Ab (610464, BD transduction). The cells were incubated with anti-rabbit or anti-mouse IgG secondary antibodies conjugated with Alexa Fluor 594 or 488 (Molecular Probes), respectively. Fluorescent images were captured with an IX51 microscope (Olympus; Tokyo, Japan) and DP70 Digital camera (Olympus).

## Competing interests

The author(s) declare that they have no competing interests.

## Authors' contributions

DS carried out almost all of the experiments, helped conceive the study and drafted the manuscript. MS performed FACS analysis. HN conceived the study, reviewed and analyzed all data and drafted the manuscript. All authors read and approved the final manuscript.

## Supplementary Material

Additional file 1 Activation of GATA-dependent reporters by GATA6 or GATA4 in ES cells. Activities of *pGata6-luc *and *pFgf3-tk-luc *in ES cells carrying various inducible GATA expression units with or without induction. Click here for file
