# Supplementary material for: Extra-embryonic endoderm cells derived from ES cells induced by GATA Factors acquire the character of XEN cells
Source: BMC Dev Biol. 2007 Jul 3;7:80. doi: 10.1186/1471-213X-7-80 (PMC1933422; doi:10.1186/1471-213X-7-80)
Supplement: Additional file 1 — Activation of GATA-dependent reporters by GATA6 or GATA4 in ES cells. Activities of pGata6-luc and pFgf3-tk-luc in ES cells carrying various inducible GATA expression units with or without induction. [file 1471-213X-7-80-S1.doc]

**Additional file 1.**

**Activation of GATA-dependent reporters by GATA6 or GATA4 in ES cells.**

Activities of *pGata6-luc* (A) and *pFgf3tk-luc* (B) in ES cells carrying various inducible GATA expression units with (exo-Gata ON) or without (OFF) induction. *pGata6-luc* contains the genomic sequence 11052529-11054393 on Chromosome 18 in the Ensembl mouse genome database, which covers the promoter element and the first intron with putative GATA binding sites. For the construction of *pFgf3-tk-luc*, three tandem PS4A (3xPS4A) sequences derived from the enhancer region of *Fgf3* containing GATA binding site [42], was generated using a pair of oligonucleotides, 5’-(gatccAAGGCTCTGTGACTCTATTGTCTCTGCTCCTATCTGTGCa) -3’, which include a BamHI site (underlined) and 5’-(gatctGCACAGATAGGAGCAGAGACAATAGAGTCACAGAGCCTTg) -3’, which include a BglII site (underlined). After annealing these oligonucleotides, these fragments were ligated by T4 DNA ligase, and were complete digested by BamHI and BglII. After 8% poly-acrylamide gel electrophoresis, 3xPS4A were cutting out from the gel and were filled-in with dGTP and dATP. 3xPS4A were ligated into XhoI sites partially filled-in with dTTP and dCTP of *ptk-Luc*, upstream of the minimal HSV-*thymidine kinase (tk)* promoter, resulting in the generation of *pFgf3-tk-Luc*. The inserts were sequenced using an ABI 3130xl genetic analyzer. These reporters as well as *pGL3* and *ptk-luc* as control were transfected into ES cells with *pCMV-RL* by lipofection followed by culture with or without Dex (for 5G6GR11-2 and 5G4GR4-3) or Tc (for EBRTc-G6 and SKG612) for 24 hours, and cell lysates were prepared for dual-luciferase assay. Both reporters were significantly activated by induction of GATA6GR or GATA4GR, suggesting they might be directly regulated by GATA factors. Tc-dependent activation was not observed in this experiment but we suppose it is due to slow kinetics of activation of GATA factors in this system, which require both transcription and translation to generate active GATA factors, whereas GATA-GR does not need both.
